# Structural Evaluation and Conformational Dynamics of *ZNF141^T474I^* Mutation Provoking Postaxial Polydactyly Type A

**DOI:** 10.3390/bioengineering9120749

**Published:** 2022-12-01

**Authors:** Yasir Ali, Faisal Ahmad, Muhammad Farhat Ullah, Noor Ul Haq, M. Inam Ul Haq, Abdul Aziz, Ferjeni Zouidi, M. Ijaz Khan, Sayed M. Eldin

**Affiliations:** 1 Department of Computer Science and Bioinformatics, Khushal Khan Khattak University, Karak 27200, Pakistan; 2 National Centre for Bioinformatics, Quaid-I-Azam University, Islamabad 44000, Pakistan; 3 Biology Department, Faculty of Arts and Sciences of Muhayil Aseer, King Khalid University, Abha 62529, Saudi Arabia; 4 Department of Mathematics and Statistics, Riphah International University I-14, Islamabad 44000, Pakistan; 5 Department of Mechanical Engineering, Lebanese American University, Beirut 13-5053, Lebanon; 6 Center of Research, Faculty of Engineering, Future University in Egypt, New Cairo 11835, Egypt

**Keywords:** *ZNF141* gene, point mutation, molecular dynamics simulations, non-synonymous SNPs, postaxial polydactyly

## Abstract

Postaxial Polydactyly (PAP) is a congenital disorder of limb abnormalities characterized by posterior extra digits. Mutations in the N-terminal region of the Zinc finger protein 141 (*ZNF141)* gene were recently linked with PAP type A. Zinc finger proteins exhibit similarity at their N-terminal regions due to C2-H2 type Zinc finger domains, but their functional preferences vary significantly by the binding patterns of DNA. Methods: This study delineates the pathogenic association, miss-fold aggregation, and conformational paradigm of a missense variant (c.1420C > T; p.T474I) in *ZNF141* gene segregating PAP through a molecular dynamics simulations approach. Results: In ZNF141 protein, helices play a crucial role by attaching three specific target DNA base pairs. In ZNF141^T474I^ protein, H1, H3, and H6 helices attain more flexibility by acquiring loop conformation. The outward disposition of the proximal portion of H9-helix in mutant protein occurs due to the loss of prior beta-hairpins at the C terminal region of the C2-H2 domain. The loss of hydrogen bonds and exposure of hydrophobic residues to solvent and helices turning to loops cause dysfunction of ZNF141 protein. These significant changes in the stability and conformation of the mutant protein were validated using essential dynamics and cross-correlation maps, which revealed that upon point mutation, the overall motion of the proteins and the correlation between them were completely different, resulting in Postaxial polydactyly type A. Conclusions: This study provides molecular insights into the structural association of ZNF141 protein with PAP type A. Identification of active site residues and legends offers new therapeutic targets for ZNF141 protein. Further, it reiterates the functional importance of the last residue of a protein.

## 1. Introduction

Polydactyly is a common congenital hand and foot deformity that refers to the duplication of fingers or supernumerary digits. It is a genetic disorder that commonly occurs in new infants [1]. It is characterized by abnormal joints, ligament insertion, anomalous tendons, and hypoplastic structures [2]. It ranges from a minor mildest form to complete duplication of digits, thus showing a great variety in recessive patterns of inheritance [3,4]. Polydactyly is classified into three major categories: Postaxial, pre-axil and center polydactyly. Postaxial polydactyly is further classified into types A and B. It is an autosomal dominant disease with different penetrance in a diverse population [5]. This disease is spread with a rate of incidence ranging from 1 per 531 newborn infants. Besides the above, it is more frequent in African and American peoples [6]. Kalsoom et al. (2013) reported a missense variant (c.1420C > T; p.T474I) in the *ZNF141* gene segregating PAP type A [7].

The ZNF141 protein is a member of the C2-H2 (Krüppel) family of zinc finger proteins which make up to ∼2% of all genes and thus it is the second-largest family of human genes [8]. C2-H2 domain is among four abundant domains found in zinc finger proteins, i.e., C2-H2, Lin-ll, Isl-1, Mec-3 (LIM domains), plant homeodomain (PHD), and a really interesting new gene (RING) domain [9]. A typical C2-H2 domain comprises two cysteines in one chain and two histidines in the other chain. C2H2-ZNF proteins contain an effector domain next to the zinc finger region [10]. These domains have C-x-C-x-H-x-H DNA interacting motifs that bind to specific sequences like (T/A) (G/A) CAGAA (T/G/C) and repress target gene expression [11]. ZNFs have been reported to have a role in the repression of RNA Polymerase II and III promotors and RNA binding and splicing. *ZNF141* gene has an open reading frame of 1422 bp, which encodes 474 residues long protein characterized by Kruppel associated box (KRAB) domain. The KRAB domain is subdivided into box A, box B, and ten zinc finger motifs. ZNF141 protein is expressed in the brain, kidney, lungs, liver, pancreas, placenta, spleen, skeletal muscle, and testis [12]. About 103 KRAB-ZNF genes are conserved across mammals, while 136 are conserved across primates [13,14]. This signifies the functional preferences of ZNF proteins. 

Mutations in the *ZNF141* gene were recently linked with limb development. Non-Synonymous Single nucleotide polymorphisms (nsSNPs) are among the most common SNPs within the human genome. nsSNP causes the substitution of amino acids within the protein, which may alter the structure, solubility, function, charge, and stability of the protein, resulting in changed phenotypes [15,16]. These properties make it of particular concern for experimental studies [17]. Various complex diseases in humans are associated with these nsSNPs. Bioinformatics studies have aided in the estimation of the molecular mechanisms and the possible clinical consequences of nsSNPs. Structural disposition and instability of protein upon point mutations are effectively shown through computational approaches. The time-dependent behavior of the mutant protein and wild type reveals the differences between two proteins upon single amino acid substitution. The solvent accessibility and hydrogen bond estimation give insights into the conformational changes that affect proteins’ globularity. 

Bioinformatics studies have been phenomenal in Genome-wide studies (GWAS) to identify mutations [15,18], novel drug targets, novel drugs [18,19,20,21] and influence of neighbor proteins on protein function [22]. This study explored the conformational transitions, and misfold aggregation and linked the pathogenic association of missense mutation (c.1420C>T; T474I) with postaxial polydactyly type A, using structural bioinformatics approaches. This study unravels the causes of ZNF141 protein dysfunction by providing structural shreds of evidence of the protein’s behavior upon point mutation.

## 2. Methodologies

### 2.1. SNP’s Annotation

ZNF141 protein fasta sequence was retrieved from the UniProt database (https://www.uniprot.org/ accessed on 10 October 2022) through the ID: Q15928. The variations present in ZNF141 were retrieved from ENSEMBL release 95 (https://asia.ensembl.org accessed on 10 October 2022) [23]. The canonical transcript was considered to investigate nonsynonymous coding regions SNPs. Sorting Intolerant from Tolerant (SIFT) and PolyPhen v2 were used to find the impact of SNPs on protein. SIFT is a web server used to determine the effects of amino acid substitution on the protein structure and function that gives an output score in the range of 0-1; the score in 0 corresponds to tolerated while score in the range of 0.5 indicates the harmful effect of nsSNPs [24], Polymorphism Phenotyping v2 score ranges from 0.0 (tolerant) to 1.0 (deleterious). Variants with a score of 0 are predicted to be benign. Values near 1.0 are more confidently anticipated to be deleterious [25]. PROVEAN [26], ReveL [27], CADD [28] and MetaL [29] were also employed to find the functional consequences of variant ZNF141^T474I^. InterPro Server was utilized to annotate the ZNF141 protein [30].

### 2.2. ZNF141^WT^ 3D Structure Prediction

The protein multi-template structure modeling technique was employed to model the consistent and accurate ZNF141^WT^ protein structure using the Modeller v 9.25 tool [31]. Four PDB structures (5V3G, 5V3J, 5V3M, and 5WJQ) ranked according to the Global Model Quality Estimate (GMQE) and Quaternary Structure Quality Estimate (QSQE) were utilized to model the best possible structure of the protein [32]. Python scripts for model refinement were used to pre-refine the modeled structure [31].

### 2.3. Protein Model Refinement and Validation

The predicted protein model was minimized using the GROMOS 54a7 force field in Gromacs v5.1.4 [33]. Ramachandran plot was generated with PROCHECK to check the Phi and Psi angles [34]. WinCOOT v0.9.2 was used to correct the outliers, and unusual rotamers manually and remove the discontinuities and large variance in atoms B factor [35]. The refined structure was validated by ERRAT [36] and RAMPAGE tools [37]. After validating the modeled structure, the active site residues, and legend binding pockets were identified in the modeled protein’s structure using the COACH server [38].

### 2.4. ZNF141^T474I^ Structure Prediction and Comparison with the ZNF141^WT^ Structure

The mutant 3D structure was obtained from ZNF141^T474I^ protein using the amino acid swap technique in Chimera v1.15 [39]. The structure was then cleared to remove any possible clashes. Before drawing the comparison, the mutant structure was minimized with consistent configuration through GORMOS 54a7 force field in Gromacs [40]. The wild-type and mutant protein structures were then compared by the PyMol tool [41] to show the structural differences in the side chains of the two amino acids. Hope Server was used to draw a residue-to-residue comparison [42].

### 2.5. Conservation Analysis 

The ConSurf web server (http://consurf.tau.ac.il accessed on 10 October 2022) examines the evolutionary trend of the macromolecule’s amino/nucleic acid sequences to identify sections that are significant for structure and/or function. The server automatically selects homologues from a query sequence or structure, infers their multiple sequence alignment, and reconstructs a phylogenetic tree that depicts their evolutionary relationships. These data are then utilized to estimate the evolutionary rates of each sequence position within a probabilistic framework. ConSurf provides the ability to homology-model query proteins, predict the secondary structure of query RNA molecules from sequence, see the biological assembly of a query (in addition to the single chain), and map the conservation grades onto 2D RNA models.

### 2.6. Molecular Dynamics Simulations

Protein MD simulations were done for 100ns to gain structural insights into ZNF141 protein using GROMACS v5.1.4 utilizing GROMOS 54a7 force field [33]. The mutant and ZNF141^WT^ models were placed in a dodecahedral box under explicit solvent and periodic boundary conditions to perform the MD simulation. The solvated water system was further neutralized by adding Chlorine ions. Energy minimization was done to have Fmax <1000 kj/moL/nm employing the steepest descent algorithm for 5000 steps to obtain stable conformations. Both the systems were equilibrated with canonical ensembles (NVT) and isobar isothermal ensembles (NPT) with a constant temperature of 300 K and a constant pressure of 1 atm for 300 ps. The MD simulation configuration was set to run at 300 K temperature for 100 ns time. The Root mean square deviation (RMSD), Root mean square fluctuation (RMSF), radius of gyration (Rg), solvent accessible surface area (SASA), and Hydrogen bond Analysis was performed utilizing different Gromacs modules. Protein PDB files were generated after 100 ns simulation and analyzed through the PDBsum tool [43]. The chimera Residue to residue (RR) distance map tool was used for RR distance and standard deviation comparison of initial and final structures [44].

## 3. Results

### 3.1. SNPs Annotation

Variants in the *ZNF141* gene were retrieved from the ENSEMBL database. Non-synonymous SNPs were selected out of all variants. There were 272 missense variants of which 145 were coding sequence variants and among them, only one SNP (rs587776959; T474I) was found to have clinical significance as pathogenic. Different tools with different logical algorithms were used for the structural and functional annotation of the SNP. SIFT predicted the nsSNP as deleterious with a score of 0.01. Polyphen2 also indicated the nsSNP as harmful with a score of 0.997. The functional impact of the nsSNP on protein function predicted by PROVEAN was also deleterious, with a score of −4.810. The CADD score was 17, the ReveL score was 0.119 and the MetaLR score was 0.046 (Supplementary Table S1).

### 3.2. Structure Prediction of C2-H2 Domain (171-474) of ZNF141 Protein

The InterPro server annotated the protein as a member of the C2-H2 (Kruppel) family of Zinc finger proteins having ten Zinc finger motifs and Kruppel-associated box domain (KRAB) (Supplementary Figure S1), which is further divided into box A and box B. Since, the mutation was mapped at the end of C2-H2 Domain, the 3D structure of C2-H2 domain (Res; 171–474) of ZNF141 protein of human was predicted by Modeller against multiple templates having PDB ID’s: 5V3G, 5V3J, 5V3M and 5WJQ. 

### 3.3. Validation of 3D Structure

**Figure 1:**
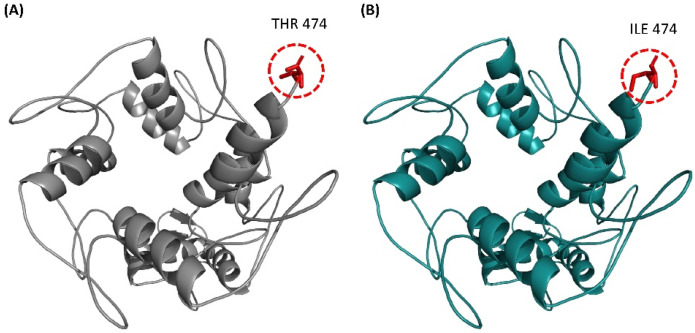
3D structure of C2-H2 (Kruppel; res 171–474) domain of ZNF141^WT^ and ZNF141^T474I^ protein with the residual position of the SNP; T474I mapped on it. (**A**) 3D structure of C2-H2 domain with Thr474 encircled and colored in red. (**B**) 3D structure of mutant C2-H2 domain with Thr474 encircled and colored in red. All the loops are represented in yellow color and helices in magenta.

The predicted 3D structure (Figure 1A) was validated by different computational tools that use different protein properties for structure validation. Ramachandran plot shows that 96.6% of residues fall in the most favored regions and 3.31% residues in the allowed area while no residues fall in the outlier region (Supplementary Figure S2). ERRAT calculated the Quality factor as A: 92.49 (Supplementary Figure S3). COOT omega angle distortion analysis depicted no unexpected peptide bond and no unusual rotamers were found in rotamers analysis.

### 3.4. Structural Comparison of ZNF141^WT^ and ZNF141^T474I^

The mutant T474I structure was modeled and then aligned with the ZNF141^WT^ structure to show the differences between the residues. As evident in Supplementary Figure S4, potential energies for both structures show that both structures were best minimized before comparison. The structural comparison depicted the differences in the side chains of the residues. The residue-to-residue comparison elucidated that the mutant Isoleucine residue is bigger than the wild-type threonine residue, which leads to bumps (Supplementary Figure S6a). The mutant Isoleucine residue is water repellent, thus more hydrophobic than the wild-type residue. Exposure of hydrophobic residues to solvent results in the loss of hydrogen bonds and disturbs correct foldings (Supplementary Figure S6b).

### 3.5. ConSurf Conservation Analysis

**Figure 2:**
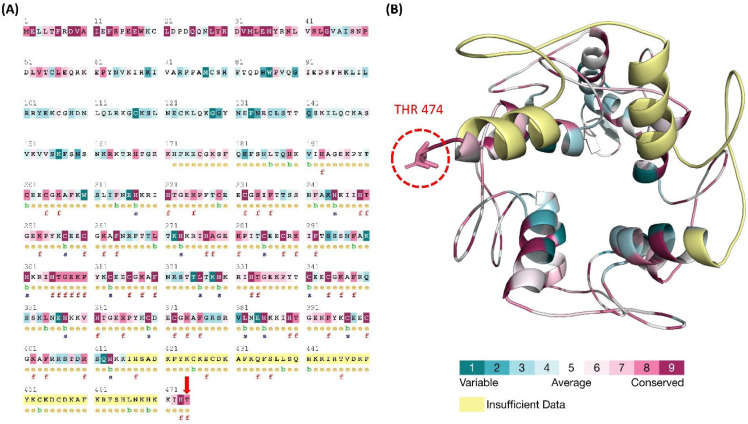
ConSurf Conservation analysis of each residue of the ZNF141 protein to show the conservation score. (**A**) Conservation score and structural and functional impact classification of each residue of the ZNF141 protein. (**B**) Cartoon representation of the ZNF141 protein in line with the color code of the conservation score.

A disease-causing mutation is often found in highly conserved locations. The conservation analyses of PAP type A promoting mutation of ZNF141 protein was performed based on protein structure. Through homologous sequence alignment with the SWISS-PROT, UniProt, and UniRef90 protein databases, the substituted Threonine residue was determined to be in a highly conserved amino acid region. The conservation score was given an 8 out of a possible 9. Additionally, the 474th Threonine residues was also predicted to have a functional impact on ZNF141 protein. Figure 2 depicts the key findings of the conservation analysis.

### 3.6. Molecular Dynamics Simulation Analysis

#### 3.6.1. Stability of ZNF141 Protein

**Figure 3:**
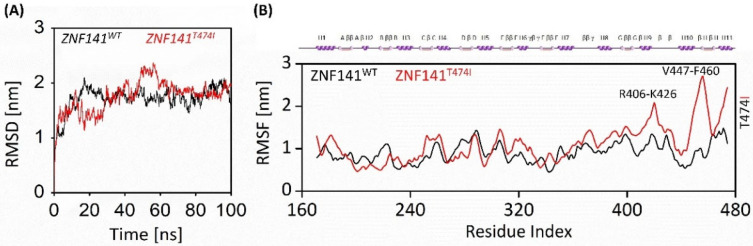
Post MD simulation analysis of ZNF141^WT^ and ZNF141^T474I^ proteins along the course of 100ns MD simulation. (**A**) RMSD of wt-ZNF41 and variant T474I represents the overall instability of the ZNF141^T474I^ (**B**) RMSF plots for wt-ZNF41 and variant ZNF141^T474I^. Residues R406-K426 and V447-F460 have shown large variations in terms of flexibility. The secondary structure elements are also shown over the RMSF plot. The black color indicates ZNF141^WT^, and the red color is for mutant T474I.

Molecular dynamics simulations of 100 ns revealed the disruptive effect of mutant T474I compared to the ZNF141^WT^. We did not find significant structural changes in wild-type protein as the RMSD was relatively stable after 30 ns except for the loops connecting the zinc fingers, which were flexible during simulation. Consequently, the RMSD value of ZNF141^WT^ closed at 1.62 nm, with an average of 1.71 nm through 100 ns simulation. On the contrary, the T474I variant was unstable with more obvious fluctuations in RMSD value. The RMSD value of variant T474I closed at 1.9 nm with a mean value of 1.76 nm, much higher than that of ZNF141^WT^ protein. These findings suggest that the wild-type ZNF141 protein is more stable than the mutant type (Figure 3A). 

#### 3.6.2. Flexibility Analysis of ZNF141 Protein 

The residual flexibility was shown by RMSF analysis as Figure 2C manifests threonine fluctuations with substituted isoleucine residue. The mutation affected the residual flexibility at position 474 along with the overall flexibility of the protein. The RMSF of wild-type Threonine residue was noted as 1.17 nm, while mutated Isoleucine was recorded as 2.39 nm. This is particularly because of the mutation at this point. The transition of parts of H1, H3, H6, and H7 helices into loops increases the overall flexibility of the protein from 0.91 nm to 1.11 nm. This transition might be supported by instability caused due to unwrapping of the Zinc fingers besides the substitution of Threonine. Besides the collective increase, the RMSF in the proximity of T474I (res 470–474) was much higher than ZNF141^WT^ (Figure 3B). 

#### 3.6.3. Gyration Analysis of ZNF141 Protein

**Figure 4:**
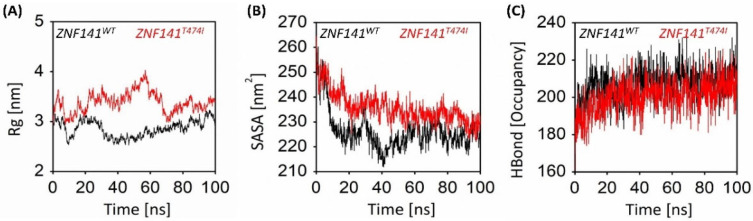
Radius of gyration, Solvent accessible surface area, and Hydrogen bond analysis for ZNF141 protein shows a significant correlation between gyration and solvent accessible area. (**A**) The radius of Gyration analysis for wt-ZNF41 and mutant T474I through the course of 100 ns molecular dynamics simulation. (**B**) The solvent-accessible surface area of wt-ZNF141 protein after 100 ns simulations. (**C**) Hydrogen bond occupancy shows that the number of intra-hydrogen bonds is showing an increasing trend through simulation time.

The gyration analysis manifested the change in the overall compactness of the mutant T474I compared to the ZNF141^WT^ structure (Figure 4A). The wild-type protein was more compact with an Rg score of 2.86 nm than the mutant protein having an Rg score of 3.39 nm, indicating a significant decrease in the globularity of protein. After the simulation time, the ZNF141^WT^ shows compactness of 2.9 nm while T474I shows 3.4 nm, reflecting that mutant T474I protein exhibited more variations. Interestingly, the Rg value of mutant T474I at the start of the simulation was 2.93 nm which remained higher up to 4.02. This indicates that the mutant protein has lost compactness which may result in protein dysfunction. 

#### 3.6.4. Solvent Accessible Surface Area Analysis

Solvent accessible surface area (SASA) analysis was performed to investigate the hydrophobic core regions of wild-type and mutant protein. Significant changes in SASA were observed for both proteins (Figure 4B). The average SASA value for wild-type ZNF protein was 226.19 nm^2^ while mutant protein was 235.66 nm^2^ (Table 1). The SASA values vary significantly because of the unwinding of protein folds, making the surface exposed to solvent (Figure 3B,C). This leads to the exposure of hydrophobic residues to the solvent, subsequently unfolding the protein and causing protein dysfunction.

#### 3.6.5. Intra-Protein Hydrogen Bond Analysis

Hydrogen bond analysis provides an essential understanding of the intramolecular hydrogen bond network of ZNF141^WT^ and ZNF141^T474I^ variants. Figure 4C provides insights into the H-bond network of both models. Careful evaluation of the number of hydrogen bonds with length ≤ 3.5 Å revealed that the number of hydrogen bonds with single point mutations significantly varied in the ZNF141 proteins. ZNF141^WT^ has an average of 207 H-bonds, while variant ZNF141^T474I^ recorded the average number of hydrogen bonds as 200 (Table 1). The final wild-type structure has 212H-bonds while mutant ZNF141^T474I^ has 214 at the end of the simulation. Loss in H-bonds reflects reduce stability and compactness of the structure. To check if our simulation is physically valid and there is no systematic drift, the total energies of both systems were calculated and compared through the course of the simulation. 

### 3.7. Secondary Structure Analysis

Secondary structural changes were evaluated for both the wild-type and mutant ZNF141 proteins. We noticed that mutant ZNF141^T474I^ has experienced significant changes in the secondary structure as compared to the ZNF141^WT^ structure (Supplementary Figure S5A,B). H1, H3, H6 and H7 helices were partially converted into loops region, making the protein flexible (Figure 4B). The beta hairpins were converted into loops resulting in the outward disposition of the proximal region after the H9 helix. This outward disposition was also the cause of an increase in the solvent-accessible surface area of the mutant protein. These results were consistent with results obtained from other analyses, which depicted that decreased stability and increased solvent accessibility of ZNF141 are the causes of PAP.

### 3.8. Residue to Residue Distance Map

**Figure 5:**
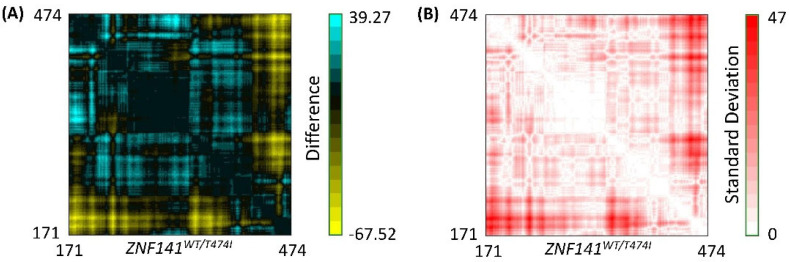
RR distance map comparison of wt-ZNF41 and mutant T474I. (**A**) RR distance map difference of wt-ZNF141 and mutant ZNF141 protein after simulation (**B**) Standard deviation of RR distance map of wt-ZNF141 and ZNF141T474I protein after 100 ns MD simulation.

RR distance map generated for the average structure of the systems showed distance-based differences and standard deviations among residues that were presented via a color-coded map as shown in Figure 5. Results deduced in the current system have been investigated for equivalent pairs by subtracting the distance of the initial structure from the next one. Herein, a minor change has been studied with a pair vise correlation of wild-type protein structure before and after simulation. An evident residual intensity has been found, indicating the stability of the system with no confirmatory changes. However, in the case of mutant protein structure, besides loop regions, significant confirmatory changes have been observed in the helix region, resulting in a drastic increase in the residue-to-residue distance. The standard deviation between final structures is shown in Figure 5B, which further delineates the conformational changes in both proteins. The open conformation phenomena drive these changes upon point mutation.

### 3.9. Dynamic Cross Correlation Map (DCCM)

**Figure 6:**
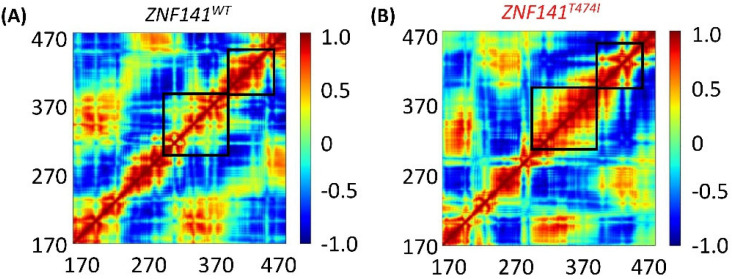
The dynamic cross-correlation map (DCCM) depicts the collective atom fluctuations for both the systems together with the associated movements of protein residues. The red hue symbolizes positive correlation, the white color represents local displacement, and the blue color represents negative correlation. (**A**) DCCM graph of ZNF141^WT^ shows significant positive and negative correlation at different positions while (**B**) shows the DCCM of ZNF141^T4741^. The correlation map of R406-K426 and V447-F460 residues is completely different for both systems.

We created and analyzed a dynamics cross-correlation matrix to explore the functional displacements of ZNF141 atoms as a function of time (DCCM). When all mutant complexes were compared to wild-type complexes, different patterns of associated movements were discovered (Figure 6). However, the correlation of atomic displacements differed significantly in ZNF141^WT^, whereas mutant ZNF141^T474I^ displayed differently correlated movements (Figure 6B). T474I had weak, negatively linked movements from residues R406-K426 and V447-F460, although it had partial correlations between residues of themselves that are opposite to the correlation observed for the same residues of ZNF141^WT^. Helix 9 of ZNF141^T474I^ displayed distantly linked motions than the wild-type protein. The movement of loops varied between protein models, which corresponds to the relevant RMSF (Figure 2) Helix 1 and 3 showed comparable linked motions in both systems. In summary, the mutant had different correlated movements than the ZNF141^WT^ protein, where most of the residues showed negative correlations.

### 3.10. Essential Dynamics of ZNF141 Protein

**Figure 7:**
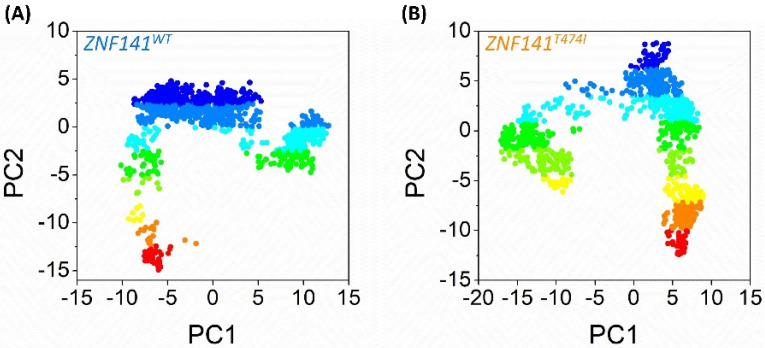
Both systems’ principal component analysis (PCA). The scattered plots (**A**,**B**) for the ZNF141WT and ZNF141T474I exhibit motion projection by showing the first two eigenvectors (PC1 vs. PC2) acquired from each system’s full 100 ns trajectory. The color gradient depicts the shift of eigenvectors from dark red to blue during MD simulations.

These findings may be supported by examining the graphical depiction of total system mobility along PC1 and PC2, which allows us to investigate the direction and amount of the motions that contribute to total system mobility. According to the projections, the WT system moves in the other way, causing an uneven expansion of the structure (Figure 7), which is consistent with Rg findings; that is, R406-K426 and V447-F460 areas moved in the opposite direction with greater amplitude. However, in comparison to WT, the direction of movement changes for ZNF141^T474I^, and the amplitude of movement is greater for the C2-H2 area and specifically R406-K426 and V447-F460 (Figure 7B), which correlates to the cluster distribution of PC1 versus PC2 projection. This mutation not only enhances the flexibility of the C2 H2 domain but also alters its orientation, potentially increasing deformation and altering the protein structure.

### 3.11. Identification of Ligand, Active Sites Residues, and Enzyme Commission Number

COACH server predicted the active site residues that were possibly the sites of attachment for the ligand. The ligand ZN has the highest C-score of 0.17 among all the predicted ligands and the residues with the highest probability of being active site residues were Arginine 378 (Table 2). The EC number of ZN is EC 7.2.2.12.

## 4. Discussion

Post Axial Polydactyly (PAP) type A is mainly autosomal dominant, the rare anomaly of fifth digit duplication in hands and/or feet. Autosomal recessive inheritance of PAP type A has been linked with mutations in the *ZNF141* gene at different chromosomal locations. ZNF141 protein is a member of the C2-H2 type Zinc finger proteins family, which contains two cysteines and two histidines in two separate chains coordinated by a zinc ion [8]. Classical zinc finger domains have two β-sheets and one α-helix as revealed by crystallographic studies [10]; however, non-classical types of Zinc finger proteins have different combinations of C2-H2, C2-CH, and C2-C2. C2-H2 domain is among four abundant domains found in zinc finger proteins, i.e., C2-H2, Lin-ll, Isl-1, Mec-3 (LIM domains), plant homeodomain (PhD), and a really interesting new gene (RING) domain [9].

The C2-H2 Zinc finger contains a more prominent transcription factor with C-x-C-x-H-x-H motifs that interact with DNA sequences. Some C2-H2 members like ZNF217 include multiple domains that bind to specific DNA sequences, consequently repressing the gene expression in target genes [11]. The zinc finger domain collocates three base pairs of target DNA to its α-helices. The specificity of DNA sequence recognition depends upon the amino acid residues at the contact site. So, any change in these amino acids can change the binding specificity of three base pairs of DNA [45]. In addition, ZNF141 contains ten ZNF motifs, and each one of these zinc finger motifs binds to a different set of DNA sequences which, upon exploitation, results in unbinding of DNA sequences [45], causing dysfunction of protein. Previously, another C2-H2 type Zinc finger protein KLF4 was reported to have a role in keratinocyte differentiation, expressed in the suprabasal layers of the epidermis. It regulates the expression of keratinocyte differentiation genes like SPINK5, ECM1, CDSN, LCE3, and FLG. KLF4 protein’s ectopic expression accelerates the differentiation process in the epidermis, which results in epidermal barrier formation [46]. Mutation at the N-terminal region of ZNF141 has been recently linked with abnormal limb development; however, the structural basis of this association was still unknown [7]. Bioinformatics studies have been phenomenal in Genome-wide studies (GWAS) to identify mutations [19,47], their annotation [15,48,49], and impact [16], identification of novel and potent drugs [50,51,52] and its targets [18]. In silico methods are significant to define the position of a gene, predict its transcripts and interaction with neighbors [22], and determining the function and structure of a protein generated from that gene within the cell. In silico study also supports us to differentiate the neutral and deleterious SNPs through various algorithms and accessible information in the databases [52,53]. Compared to small molecule inhibitors, peptide inhibitors have less toxicity and better target selectivity. Bioinformatics analysis adds valuable insights into the macromolecular structural-functional correlation using molecular dynamics simulation of wild-type and mutant models under explicit conditions. Several studies have been conducted in the past that emphasized computational tools, especially molecular dynamics simulations that diversified the understanding of SNPs causing disease in different proteins [53].

In this study, we have established a structural association between the dysfunction of ZNF141 protein that provokes postaxial polydactyly, as reported by [7]. All 100 ns of all atoms MD simulation manifested time-dependent behavior of wild-type and mutant ZNF141 protein. The structural investigation revealed that ZNF141 protein binds to the target DNA sequence through its alpha-helix; however, the mutation T474I disrupts the structure of ZNF141 protein, subsequently resulting in partial transition of H1, H3, H6, and H7 helices into loops. Additionally, beta-hairpins in the structure disappear resulting in the flexibility of the structure. Several hydrogen bonds were lost in the mutant protein as the mutant protein lost its compactness. Solvent accessible surface area increased drastically due to point mutation, resulting in the exposure of hydrophobic residues to the solvent, causing protein malfunctioning. Secondary structure analysis shows that three beta hairpins are lost along with the partial transition of helices into loops, resulting in a loss of protein stability. The wild-type ZNF141 protein maintains the most rigid and conformationally stable zinc-bound configuration when compared with the diseased counterparts. These significant changes in the mutant protein’s stability and conformation were confirmed using essential dynamics and cross-correlation maps, which revealed that after point mutation, the overall motion of the proteins and their correlation were completely different, resulting in Postaxial polydactyly type A. Besides providing the structural basis of ZNF141 association with Postaxial polydactyly, this study also emphasizes the role of the last residue of the chain in the correct folding of the protein, its stability, and flexibility. The active site residues and ligand identification of the *ZNF141* gene provide the basis for novel gene therapies and targets for drug discovery strategies to restore the standard functionality of the ZNF141 protein.

## 5. Conclusions

The characterization of disease-associated SNP’s has a crucial role in modern genetic analysis, gene association studies, protein structure, and stability. We have investigated the structural impact of point mutation on the *ZNF141* gene using a molecular dynamics simulation assay. We have concluded that H1, H3, H6, and H7 α-helices play a crucial role in DNA binding. The loss of beta hairpins leads to bumps, loss of stability, compactness, and hydrogen bonds that results in incorrect foldings and exposure of hydrophobic residues to solvent. Subsequently, it causes protein dysfunction leading to postaxial polydactyly type A during fetal development. Altogether this study shows the structural association of ZNF141^T474I^ with PAP and may contribute to the inventory of novel inhibitors that can compete with DNA for binding based on intrinsic properties.

## Figures and Tables

**Table 1:** The calculated parameters for all the systems were obtained after 100-ns MD simulations.

S.No.	Protein	RMSD (nm) (Average)	RMSF (nm) (Average)	RMSF (T474I)	H-Bonds (Average)	Rg (nm)	SASA (nm^2^) (Average)
1	wt-ZNF141	1.71	0.91	1.17	207	2.9	226.19
2	T474I	1.76	1.11	2.39	200	3.4	235.66

**Table 2:** Ligands and binding residues of human ZNF141 protein.

Rank	C-Score	Cluster Size	PDB Hit	Lig Name	Binding Residues
1	0.17	14	2lt7A	ZN	257,260,273,277
2	0.05	6	1a1iA	Nuc.acid	337,346,348,350,353,356,357,360,374,376,377, 378,381,385,388,402,406,409,412
3	0.04	4	3g0bD	NAG	229,232,236,245
4	0.04	3	2wbsA	ZN	285,288,301,305
5	0.03	3	2i13B	Nuc.acid	337,346,348,350,353,356,357,374,376,377,378, 381,385,388,402,404,406,409,412,413,416
6	0.03	3	1tf6A	Nuc.acid	262,264,265,266,269,273,276,292,296,297,300, 304,318,320,322,325,329,332,348,350,353,357, 360,376,378,381,385,388,391
7	0.02	2	4kx7A	ZN	313,315,316,329,334

## Data Availability

All the data regarding research work are clearly presented in the research work. Some of data are provided by special request of author.

## Supplementary Materials

The following supporting information can be downloaded at: https://www.mdpi.com/article/10.3390/bioengineering9120749/s1, Figure S1: InterPro server annotation of the ZNF141 protein showing C2-H2; IPR013087 domain from residue 171-474 with ten Zinc finger motifs; Figure S2: Ramachandran plot of ZNF141; C2-H2 domain (Res 171-474) with all the residues in Preferred and allowed regions and no residue in the disallowed region; Figure S3: ERRAT validation graph showing an overall quality score of the structure as 92.491 with the low relatively low confidence score of the error for the rest of the residues; Figure S4: The potential energy of both wt-ZNF141 and T474I models after energy minimization; Figure S5: Secondary structure analysis of (A) wt-ZNF141 and (B) mutant T474I protein after 100ns molecular dynamics simulations; Figure S6: (**a**) The figure shows the schematic structures of the original (left; threonine) and the mutant (right; isoleucine) amino acid. The backbone, which is the same for each amino acid, is colored red. The side chain, unique for each amino acid, is colored black; (**b**) Superimposition of wild-type and mutant-type residues at 474th position. The wild type of threonine is shown in sticks while mutant Isoleucine is shown in Lines; Table S1: Functional impact of ZNF141 T474I mutation predicted by different tools.
