# Supplementary material for: Structural Evaluation and Conformational Dynamics of ZNF141T474I Mutation Provoking Postaxial Polydactyly Type A
Source: Bioengineering (Basel). 2022 Dec 1;9(12):749. doi: 10.3390/bioengineering9120749 (PMC9774408; doi:10.3390/bioengineering9120749)
Supplement: Supplementary file 1 [file bioengineering-09-00749-s001.zip › bioengineering-2011891-supplementary.pdf]

Decoding the molecular basis of Postaxial polydactyly type-A promoted by ZNF141 gene; an eScience view

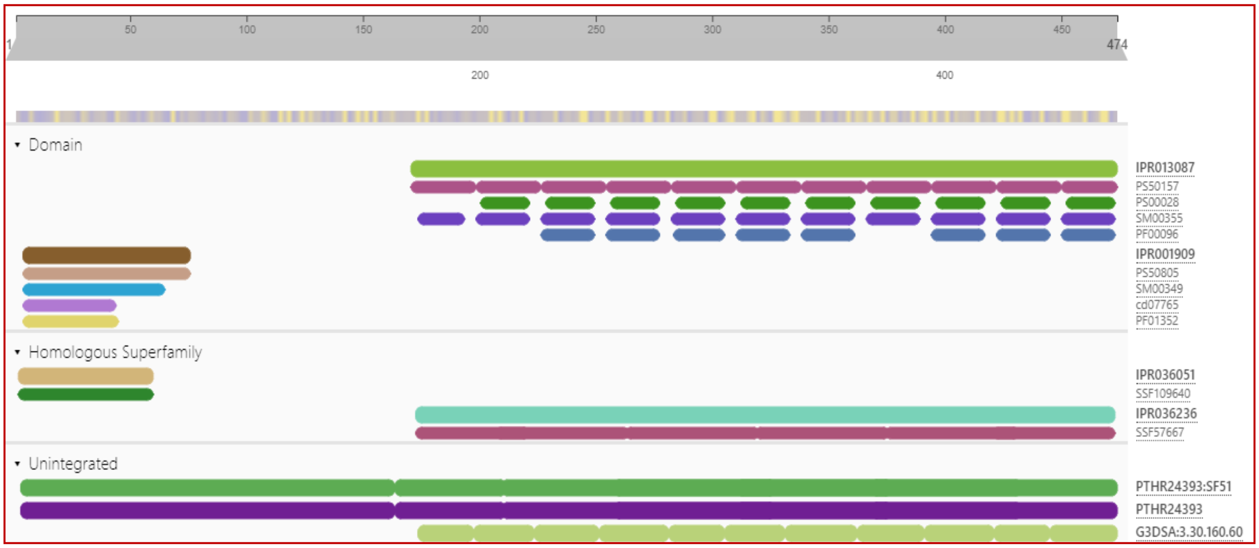

Supplementary Figure S1: *InterPro server annotation of the ZNF141 protein showing C2-H2; IPR013087 domain from residue 171-474 with ten Zinc finger motifs.*

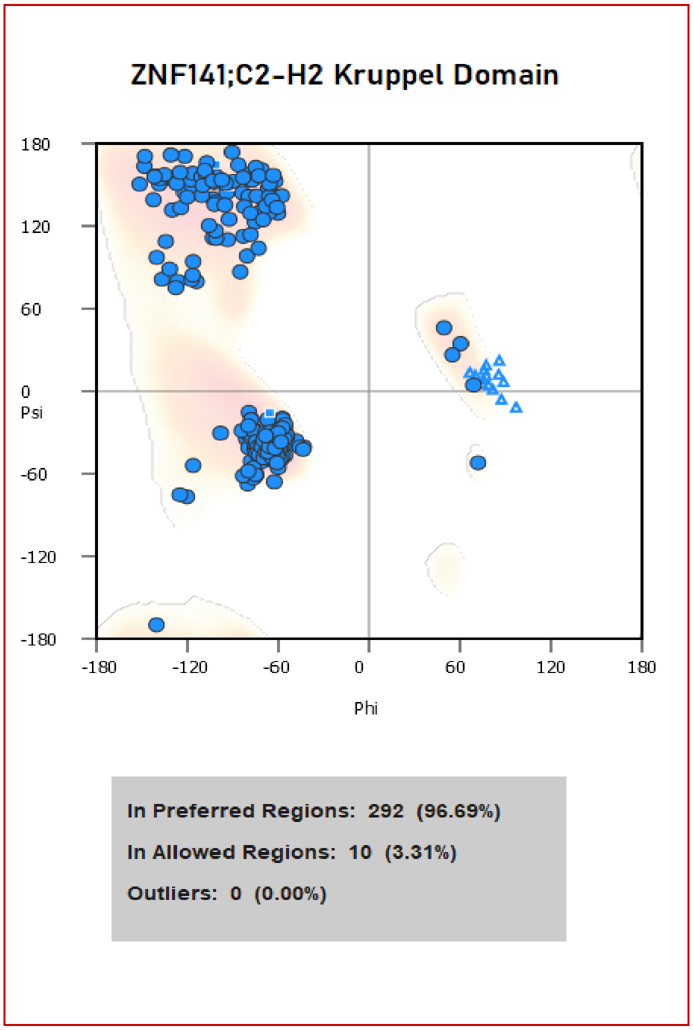

Supplementary Figure S2: *Ramachandran plot of ZNF141; C2-H2 domain (Res 171-474) with all the residues in Preferred and allowed regions and no residue in the disallowed region.*

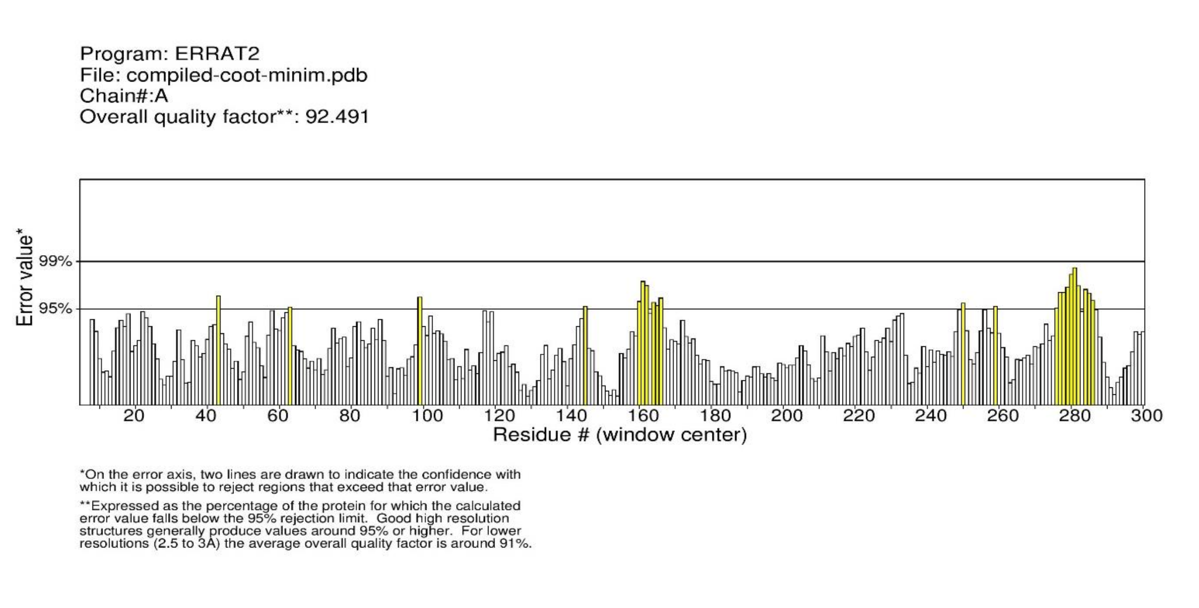

Supplementary Figure S3: *ERRAT validation graph showing an overall quality score of the structure as 92.491 with the low relatively low confidence score of the error for the rest of the residues.*

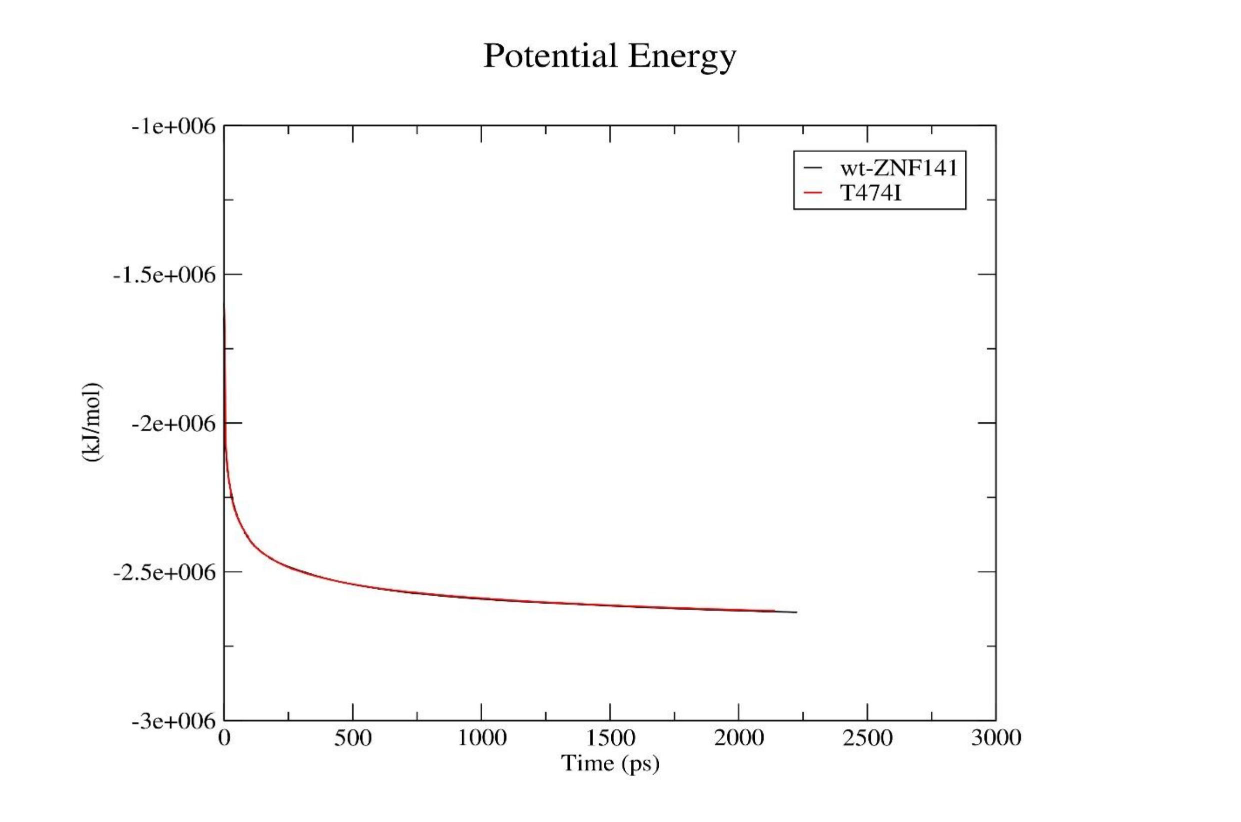

Supplementary Figure S4: The potential energy of both wt-ZNF141 and T474I models after energy minimization.

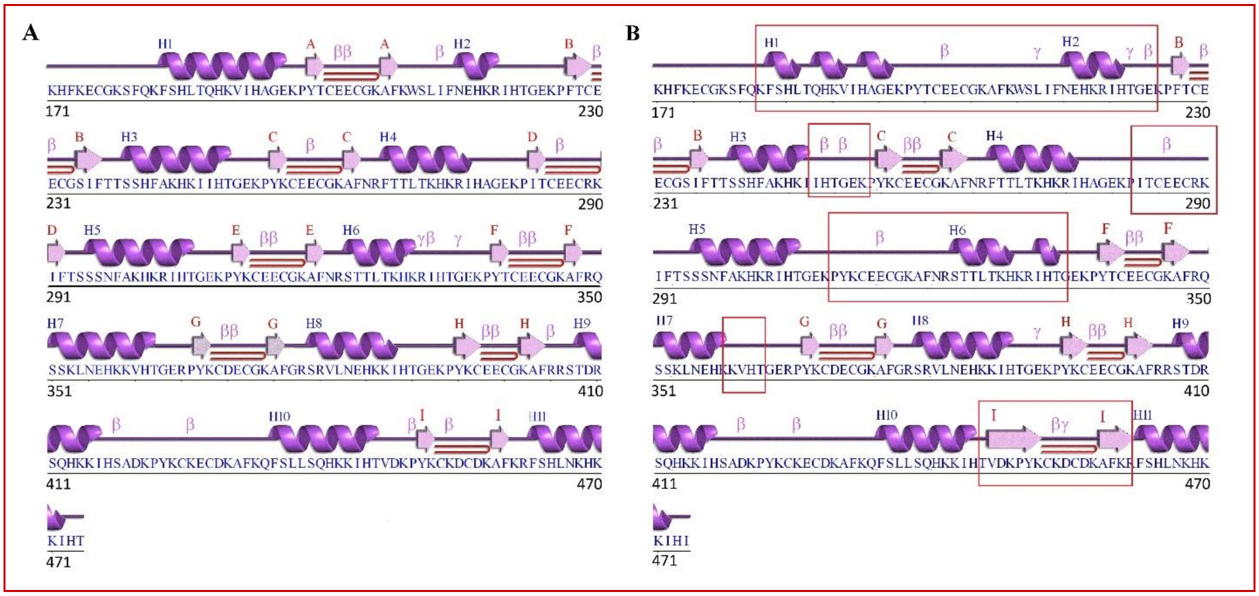

Supplementary Figure S5. Secondary structure analysis of (A) wt-ZNF141 and (B) mutant T474I protein after 100ns molecular dynamics simulations.

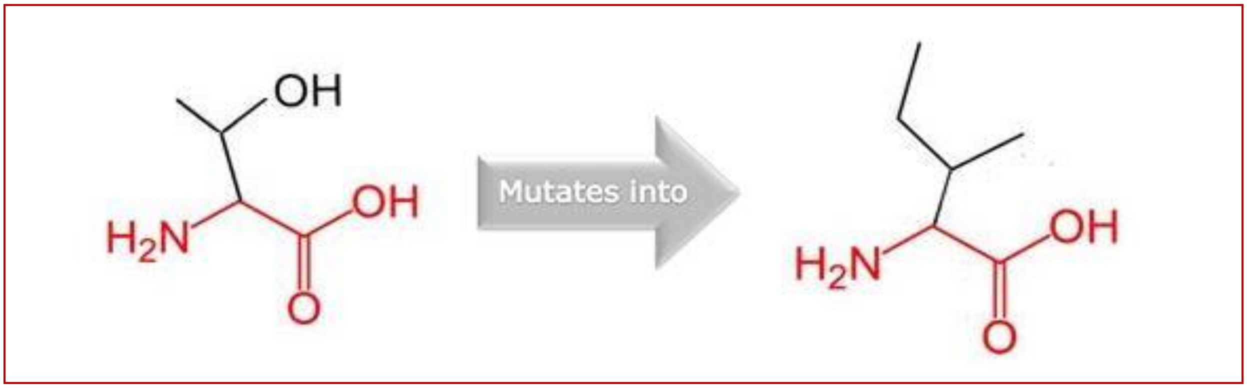

Supplementary Figure S6 (a): *The figure shows the schematic structures of the original (left; threonine) and the mutant (right; isoleucine) amino acid. The backbone, which is the same for each amino acid, is colored red. The side chain, unique for each amino acid, is colored black.*

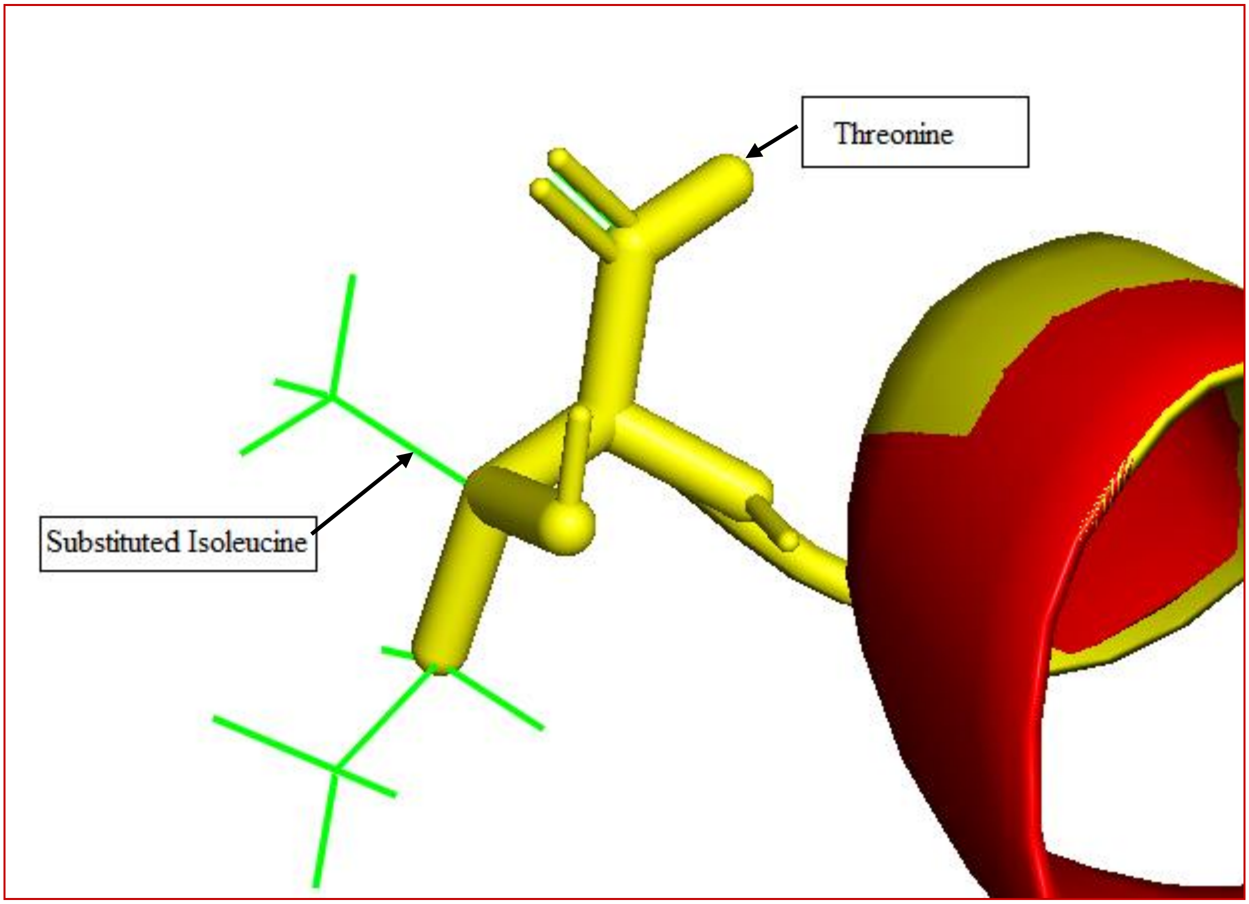

Supplementary Figure S6 (b): *Superimposition of wild-type and mutant-type residues at 474th position. The wild type of threonine is shown in sticks while mutant Isoleucine is shown in Lines.*

Supplementary Table S1: *Functional impact of ZNF141 T474I mutation predicted by different tools*

**Variant ID**	**Genomic substitution**	**A.A.**	**Location**	**SIFT**	**Polyphon**	**Revel**	**CADD**	**MetaLR**
rs587776959	C>T	T>I	474	0.01	0.998	0.119	17	0.046
